# Efficiency of Phage φ6 for Biocontrol of *Pseudomonas syringae* pv. *syringae*: An in Vitro Preliminary Study

**DOI:** 10.3390/microorganisms7090286

**Published:** 2019-08-23

**Authors:** Larindja A. M. Pinheiro, Carla Pereira, Carolina Frazão, Victor M. Balcão, Adelaide Almeida

**Affiliations:** 1 Department of Biology and CESAM, University of Aveiro, Campus Universitário de Santiago, 3810-193 Aveiro, Portugal; 2 PhageLab—Laboratory of Biofilms and Bacteriophages, University of Sorocaba, 18023-000 Sorocaba, São Paulo, Brazil

**Keywords:** phage treatment, phytopathogenic bacteria, resistance, physico-chemical factors

## Abstract

*Pseudomonas syringae* is a plant-associated bacterial species that has been divided into more than 60 pathovars, with the *Pseudomonas syringae* pv. *syringae* being the main causative agent of diseases in a wide variety of fruit trees. The most common treatments for biocontrol of *P. syringae* pv. *syringae* infections has involved copper derivatives and/or antibiotics. However, these treatments should be avoided due to their high toxicity to the environment and promotion of bacterial resistance. Therefore, it is essential to search for new approaches for controlling *P. syringae* pv. *syringae*. Phage therapy can be a useful alternative tool to the conventional treatments to control *P. syringae* pv. *syringae* infections in plants. In the present study, the efficacy of bacteriophage (or phage) φ6 (a commercially available phage) was evaluated in the control of *P. syringae* pv. *syringae*. As the plants are exposed to the natural variability of physical and chemical parameters, the influence of pH, temperature, solar radiation and UV-B irradiation on phage φ6 viability was also evaluated in order to develop an effective phage therapy protocol. The host range analysis revealed that the phage, besides its host (*P. syringae* pv. *syringae*), also infects the *Pseudomonas syringae* pv. *actinidiae* CRA-FRU 12.54 and *P*. *syringae* pv. *actinidiae* CRA-FRU 14.10 strains, not infecting strains from the other tested species. Both multiplicities of infection (MOIs) tested, 1 and 100, were effective to inactivate the bacterium, but the MOI 1 (maximum reduction of 3.9 log CFU/mL) was more effective than MOI 100 (maximum reduction of 2.6 log CFU/mL). The viability of phage φ6 was mostly affected by exposure to UV-B irradiation (decrease of 7.3 log PFU/mL after 8 h), exposure to solar radiation (maximum reduction of 2.1 PFU/mL after 6 h), and high temperatures (decrease of 8.5 PFU/mL after 6 days at 37 °C, but a decrease of only 2.0 log PFU/mL after 67 days at 15 °C and 25 °C). The host range, high bacterial control and low rates of development of phage-resistant bacterial clones (1.20 × 10^−3^) suggest that this phage can be used to control *P. syringae* pv. *syringae* infections in plants, but also to control infections by *P. syringae* pv. *actinidiae*, the causal agent of bacterial canker of kiwifruit. Although the stability of phage φ6 was affected by UV-B and solar radiation, this can be overcome by the application of phage suspensions at the end of the day or at night.

## 1. Introduction

*Pseudomonas syringae* pv. *syringae* is a phytopathogenic bacterium responsible for bacterial canker and blast (stone and pome fruits). This bacterium infects more than 180 species of plants, among them *Citrus* sp. and *Prunus* sp. [1]. It affects all commercially grown *Prunus* species, including peach (*Prunus persica*), European plum and French prune (*P. domestica*), Japanese plum (*P. salicina*), sweet cherry (*P. avium*), apricot (*P. armeniaca*), tart cherry (*P. cerasus*), almond (*P. dulcis*), and other stone fruits [2]. *P. syringae* pv. *syringae* is also responsible for bacterial blast of orange (*Citrus sinensis*) and mandarin (*Citrus rediculate*) and black pit of orange fruits, causing economic losses worldwide [3].

*P. syringae* pv. *syringae* causes damage especially under cold and humid conditions in spring when the development and spread of the bacterial blast happens quicker and more easily, and when the shoots or fruits are damaged by wind, hail, or thorns [3,4]. The disease symptoms appear as water-soaked lesions extended to the mid-vein and to the twigs surrounding the base of the petiole. Ultimately, the leaves dry and curl, while still firmly attached, and eventually fall without petioles. The necrotic areas on twigs further enlarge and the twigs are eventually killed within three to four weeks [3,4]. This pathovar can be disseminated by wind, rain, and insects, or propagated via either infested wood or nursery stock. Equipment, such as pruning tools and mechanical harvesters, may also carry the phytopathogen from plant to plant in addition to causing entry wounds.

Currently, the management of most fruit tree diseases caused by *P. syringae* pv. *syringae* is almost unattainable, due to the lack of effective chemical or biological control measures, little available knowledge of host resistance, and the endophytic nature of the pathogen during some phases of the disease cycle [2]. The available treatments for this disease are still scarce, with the most common involving frequent spraying of the orchards with copper derivatives, in particular cuprous oxide (Cu_2_O) and/or antibiotics. However, these strategies are not completely effective. Moreover, the massive use of copper and antibiotics can promote the development of resistance in the pathogen and changes in the structure of bacterial communities [5]. One of the most promising alternatives to the use of these strategies is the use of phages.

The use of phages (viruses that infect bacteria) as biocontrol agents was hindered by the advent of antibiotics, but, in recent years, the continuous selection of bacteria resistant to antibiotics or other antimicrobial agents has led to a new emphasis on phage therapy [6,7,8,9,10,11,12]. Phage therapy has several potential advantages over the use of copper compounds and antibiotics. Phages are usually highly specific to a single species or even strain of bacteria and therefore cause less damage to the normal microflora. Phages are self-replicating as well as self-limiting, replicate exponentially as bacteria, and decline when bacterial number decreases [13,14].

Phages have been used as therapeutic or prophylactic agents to control bacterial diseases in plants and there are already some approved applications of phages in the agricultural sector [15,16,17,18]. Moreover, the isolation and characterization of new phages has been described for several pathovars of *P. syringae*, such as *Pseudomonas syringae* pv. *tomato* [19], *Pseudomonas syringae* pv. *phaseolicola* [20,21,22], *P. syringae* pv. *syringae* [23], *Pseudomonas syringae* pv. *morsprunorum* [24], *Pseudomonas syringae* pv. *porri* [25], and *P. syringae* pv. *actinidiae* [26,27,28,29,30,31]. Although Nordeen et al. (1983) isolated and characterized phages of the phytopathogen *P. syringae* pv. *syringae*, they did not evaluate the application of these phages to inactivate this bacterial pathovar [23]. Moreover, to the best of our knowledge, no studies so far evaluated the therapeutic potential of other phages to control *P. syringae* pv. *syringae.*

The aim of this study was to evaluate the efficiency of one of the best-studied and already commercially available phages, the phage φ6, to control infections by *P. syringae* pv *syringae*. To our best knowledge, phage φ6 was initially characterized with the plant-pathogenic *P. syringae* pv. *phaseolicola* HB10Y [20,32], but no inactivation studies were performed by those authors, and was not yet tested to inactivate *P. syringae* pv*. syringae*. The phage φ6 is an already genomically characterized phage, that neither codifies for integrase genes, which prevent lysogeny [33,34,35], nor for virulent factors and antibiotic/metals resistance [36], and can thus be considered as safe to be used to control bacterial infections. As one of the major concerns regarding the use of phages to control infections is the emergency of phage-resistant mutants after treatment, the development of resistant mutants of *P. syringae* pv. *syringae* to phage φ6 was also evaluated in this study. Additionally, considering that plants are exposed to environmental factors and that the viability of phages can be affected by physico-chemical factors, in this study, the influence of pH, temperature, solar radiation, and UV-B irradiation on phage φ6 stability was also evaluated in order to develop an effective phage therapy strategy.

## 2. Materials and Methods

### 2.1. Bacterial Strains and Growth Conditions

The bacterial strain *P. syringae* pv. *syringae* (DSM 21482) was purchased from Leibniz-Institute DSMZ—Deutsche Sammlung von Mikroorganismen und Zellkulturen GmmH (Braunschweig, Germany). *P. syringae* pv. *actinidiae* strains (CRA-FRU 8.43, 12.54, and 14.10) were purchased from Culture Collection of C.R.A.—Centro di Ricerca per la Frutticoltura (Roma, Italy). *Pseudomonas aeruginosa* (ATCC 27853), *Aeromonas hydrophila* (ATCC 7966), *Salmonella enterica* serovar Typhimurium (ATCC 13311 and ATCC 14028), *Escherichia coli* (ATCC 25922 and ATCC 13706), and *Vibrio parahaemolyticus* (DSM 27657) were purchased from ATCC and DSM culture collections, respectively. The other bacterial strains used in this study were isolated in previous research works from water samples collected in Ria de Aveiro (Aveiro, Portugal) [37,38].

All bacteria were grown in Tryptic Soy Broth (TSB, Roseto degli Abruzzi (Te), Italia). The bacterial strains were stored at −80 °C in 10% glycerol. Before each assay, a stock culture of each bacteria was aseptically inoculated in 30 mL of TSB and was grown during 18 h at 25 °C with orbital shaking set at 120 rpm stirring. Then, an aliquot (300 µL) of each bacterial culture was transferred to 30 mL of fresh TSB and grown during 18 h at 25 °C under orbital shaking (120 rpm). The viable cell density was approximately 10^9^ colony-forming units (CFUs)/mL.

### 2.2. Preparation of Phage φ6 and Enrichment

Phage φ6 (DSM 21518) was purchased from Leibniz-Institute DSMZ—Deutsche Sammlung von Mikroorganismen und Zellkulturen GmmH (Braunschweig, Germany). Phage φ6 is a double-stranded RNA phage and belongs to the family *Cystoviridae* [39,40]. Phage suspensions were prepared, departing from the phage stock previously prepared in SM buffer (0.1 M NaCl (Sigma, St. Louis MO, USA), 8 mM MgSO_4_ (Sigma, St. Louis MO, USA), 20 mM Tris-HCl (Sigma, St. Louis MO, USA), 2% (w/v) gelatin, pH 7.5) using *P. syringae* pv. *syringae* as the host. Three hundred microliters of the phage stock were added to thirty milliliters of SM buffer and one milliliter of *P. syringae* pv. *syringae* in the exponential growth phase. The suspension was grown overnight and incubated at 25 °C under orbital shaking set at 50 rpm. The lysate was centrifuged at 13.000 rpm for 10 min at 4 °C and the supernatant was filtered through a polyethersulphate membrane with a 0.22 µm pore size (Merck-Millipore, Darmstadt, Germany), to remove intact bacteria or bacterial debris. The phage suspension was stored at 4 °C until use was in order and the titer was determined via the double-layer agar method [41]. Successive dilutions of the phage suspension were performed in phosphate buffered saline (PBS; 137 mmol^−1^ NaCl (Sigma, St. Louis MO, USA), 2.7 mmol^−1^ KCl (Sigma, St. Louis MO, USA), 8.1 mmol^−1^ Na_2_HPO_4_·2H_2_O, 1.76 mmol^−1^ KH_2_PO_4_ (Sigma, St. Louis MO, USA), pH 7.4) and 500 μL of each dilution were added to 200 μL of fresh *P. syringae* pv. *syringae* culture, mixed with 5 mL of TSB 0.6% top agar layer (30 g/L TSB (Liofilchem, Roseto degli Abruzzi (Te), Italy), 6 g/L agar (Liofilchem, Roseto degli Abruzzi (Te), Italy), 0.05 g/L CaCl_2_ (Sigma, St. Louis MO, USA), 0.12 g/L MgSO_4_ (Sigma, St. Louis MO, USA), pH 7.4) and placed over a Petri plate containing solid Tryptic Soy Agar (TSA, Roseto degli Abruzzi (Te), Italy). The plates were incubated at 25 °C for 18 h. The results are expressed as plaque forming units per milliliter (PFU/mL).

### 2.3. Determination of the Molar Extinction Coefficient of the Isolated Phage φ6 Particles

The molar extinction coefficient of phage φ6 was determined according to [42]. Successive dilutions of phage 6 (10^8^ PFU/mL), using different volumes of the phage suspension, were prepared. After, the absorbance was determined using a spectrophotometer (model Halo DB-20, Livingston, United Kingdom) at 265 nm (wavelength producing the maximum absorption of the phage suspension) and 320 nm (wavelength where there is little light absorption from phage chromophores).

### 2.4. Phage φ6 Host Range: Spot Test and Efficiency of Plating (EOP) Assays

Phage host range was determine using the bacterial strains listed in Table 1. Phage host range was determined by spot testing according to the procedure described by [43]. Briefly, three milliliters of TSB 0.6% agar, previously inoculated with 300 μL of bacterial culture , were overlaid on solid TSA and spotted with 10 μL of the phage suspension. The plates were incubated at 25 °C and examined for the presence of lysis plaques after 18 h. Bacterial sensitivity to the phage was established by a clear lysis zone at the spot. Depending on the clarity of the spot, bacteria were differentiated according to either a clear lysis zone (+) or no lysis zone (−) (Table 1). The EOP was determined for those bacteria with positive spot tests (occurrence of a clear lysis zone), using the double-layer agar method [41]. The plates were incubated at 25 °C and examined for the presence of plaques after 18 h. The EOP for each bacterial host was calculated by comparison with an efficacy of *P. syringae* pv. *syringae* (host) (Table 1). The EOP was calculated as (average PFU on target bacteria/average PFU on host bacteria) x 100 [44,45]. The EOP value obtained with the host strain was considered as EOP = 100%. EOP values are presented in the manuscript as the mean of three independent measurements followed by their standard deviation.

### 2.5. One-Step Growth Curve

Phage φ6 suspension (final concentration of 10^6^ PFU/mL) was added to 10 mL of the bacterial culture of *P. syringae* pv. *syringae* (cell density of 10^9^ CFU/mL) in order to have a MOI of 0.001 and the resulting suspension incubated without shaking during 5 min at 25 °C [46]. The mixture was centrifuged at 10,000 rpm for 5 min, the pellet was re-suspended in 10 mL of TSB, and incubated at 25 °C. Samples (1 mL) were collected at time 0 and at time intervals of 10 min up to 150 min of incubation and immediately tittered by the double-layer agar method [41]. The plates were incubated at 25 °C and examined for the presence of plaques after 18 h. Three independent assays were performed. The results were subsequently plotted to determine the phage eclipse period, latent period, intracellular accumulation period, and burst size. The one-step growth curves data produced was better adjusted via nonlinear fitting the data to a typical sigmoidal curve (or 4-parameter logistic regression model) (Equation (1)):

(1) $$\mathrm{Log}\;\left(P_{t}\right)=\;m_{1}+\frac{m_{2}-m_{1}}{1+{\left(\frac{t}{m_{3}}\right)}^{m_{4}}}$$

where *P_t_* is the phage concentration (PFU/mL) at time *t*, *m*_1_ is the response at *t* = 0, *m*_2_ is the response at *t* = ∞, *m*_3_ is the curve inflection point, *m*_4_ is the slope that defines the steepness of the curve, and *t* is the time (min). Nonlinear fitting of the phage growth data to the model in Equation (2) was performed using the software KaleidaGraph v. 4.5.2 for MacOS X (Synergy Software, Reading PA, USA).

### 2.6. Adsorption Curve

Phage φ6 suspension (final concentration of 10^6^ PFU/mL) was added to 10 mL of the bacterial culture (final concentration of 10^9^ CFU/mL) to obtain a MOI of 0.001 [47] and the resulting suspension was incubated at 25 °C. The mixture was centrifuged at 10,000 rpm for 5 min and supernatants were immediately filtered through 0.2 µm pore-size filters (Millipore Bedford, MA, USA). The filtrates containing unadsorbed or reversibly adsorbed phage particles were diluted and titrated. The plates were incubated at 25 °C and examined for plaques after 18 h. Adsorption was expressed as the percentage decrease of phage titer in the supernatant, as compared to the time zero. Suspensions of phage without any bacterial cells were used as a no-adsorption standard for calculations [47]. Three independent assays were performed. The adsorption rate was estimated via nonlinear fitting of the experimental data to the model in Equation (2) [48,49,50], viz:

(2) $$\mathrm{Ln}\left(\frac{P_{t}}{P_{0}}\right)=-\delta \left(\frac{X_{0}}{\mu \left(t\right)}\right)\left(e^{\mu \left(t\right)\cdot t}-1\right)$$

where *P_t_* and *P*_0_ are phage concentrations at times *t* and 0, respectively, *δ* is the adsorption rate to be estimated, *X*_0_ is the concentration of (susceptible, uninfected) bacterial cells at time 0, *µ*(*t*) is the bacteria multiplication rate, and *t* is the infection time. Nonlinear fitting of the adsorption data to the model in Equation (2) was performed using the software KaleidaGraph v. 4.5.2 for MacOS X (Synergy Software, Reading PA, USA).

### 2.7. Bacterial Kill Curves

*P. syringae* pv. *syringae* (final concentration of 10^5^ CFU/mL) inactivation by the phage φ6 (final concentrations of 10^5^ and 10^7^ PFU/mL) was evaluated at MOI 1 and MOI 100. For each assay, two control samples were included: The bacterial control (BC) and the phage control (PC). The bacterial controls were inoculated with *P. syringae* pv. *syringae* but not with phage φ6, and the phage controls were inoculated with phage φ6 but not with bacterial cells. Controls and test samples were incubated under exactly the same conditions. Aliquots of test samples (BP, bacteria plus phage) and of the bacterial and phage controls were collected at time 0 and after 2, 4, 6, 8, 10, 12, 14, 18, and 24 h of incubation. In all assays, the phage titer was determined in triplicate by the double-layer agar method [41] after an incubation period of 18 h at 25 °C. Bacterial concentration was determined in triplicate in solid TSA medium via the drop-plate method after an incubation period of 48 h at 25 °C. Ten colonies of the test samples (BP), at a MOI of 1, were picked and purified by successive sub-culturing in TSA in order to remove attached phage particles. These colonies were used in further experiments (as described in Section 2.8). Three independent experiments were performed for each condition.

### 2.8. Phage Sensitivity of Surviving Bacteria after Phage Exposure

**Figure 1:**
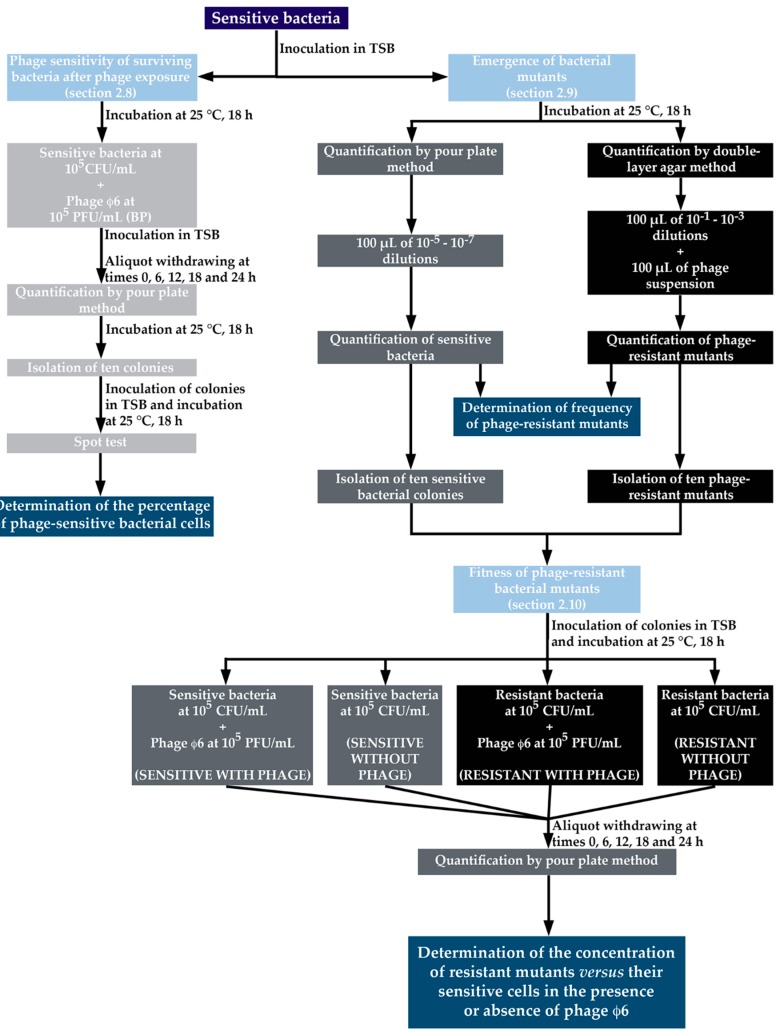
Design of the experimental work performed to test the “phage sensitivity of surviving bacteria after phage exposure” (Section 2.8), “emergence of bacterial resistances to phage” (Section 2.9), and “fitness of phage-resistant bacterial mutants” (Section 2.10). TSB: Tryptic Soy Broth.

The phage sensitivity of surviving bacteria after phage exposure was determined using the killing curves samples collected at time zero and after 6, 12, 18, and 24 h of incubation. The bacterial colonies of the test samples (BP) at a MOI of 1 were used (as described in Section 2.7.) (Figure 1), because this MOI was the best one to control *P. syringae* pv. *syringae*. To check whether the bacterial strain remained sensitive to phage φ6 during the bacterial kill assays, 10 isolated colonies (randomly picked) of the bacterial control and test samples at time 0, 6, 12, 18, and 24 h were inoculated separately into TSB and grown at 25 °C during 18 h at 120 rpm and tested using the spot test procedure. Three hundred microliters of bacterial culture previously inoculated with TSB 0.6% agar were overlaid on solid TSA and spotted with 10 μL of the phage φ6 suspension. The plates were incubated at 25 °C and examined for the presence of lysis plaques after 18 h. The percentage of phage sensitivity of surviving bacteria was calculated as (the number of sensitive bacteria (positive spot test) in test samples (BP)/the number of tested colonies)) × 100. Three independent assays were performed.

### 2.9. Isolation of Phage-Resistant Mutants and Determination of the Frequency of Emergence of Phage-Resistant Bacterial Mutants

The development of resistant mutants of *P. syringae* pv. *syringae* to phage φ6 was evaluated according to the procedure described by [51] (Figure 1). To determine the frequency of phage-resistant bacteria, 10 isolated colonies from a plate with sensitive bacteria were selected and inoculated into 10 test tubes with 5 mL of TSB, and grown at 25 °C for 18 h at 120 rpm stirring. Aliquots of 100 µL from the 10^−1^ to 10^−3^ dilutions of the bacterial culture and aliquots of 100 µL of the phage from a stock solution of 10^8^ PFU/mL were inoculated into test tubes containing TSB 0.6% agar, plated on TSA plates, and incubated at 25 °C for 48 h. Simultaneously, 100-µL aliquots of 10^−5^ to 10^−7^ dilutions of the bacterial culture were plated by incorporation on TSA plates without phage and incubated at 25 °C for 48 h. The calculation of the frequency of *P. syringae* pv. *syringae* spontaneous phage-resistant mutants was done by dividing the number of resistant bacteria (obtained from the bacteria that emerge in the presence of phage particles) by the total number of sensitive bacteria (prepared from the culture without phages) [51]. Sensitive and phage-resistant colonies were picked up and purified by successive sub-culturing in TSA in order to remove attached phage particles, and were used in further experiments (as described in Section 2.10). Three independent assays were performed.

### 2.10. Fitness of Phage-Resistant Bacterial Mutants

The concentration of the sensitive and resistant bacterial cell populations (isolated after phage exposure in Section 2.9) was quantified in the presence and in the absence of phage φ6, in order to evaluate the cost (“the fitness”) that the bacteria suffers to develop resistance to the phage (Figure 1). The MOI of 1 was selected for these experiments because it was the best condition to control *P. syringae* pv. *syringae*. Sensitive *P. syringae* pv. *syringae* was added to 2 out of 4 samples in order to obtain a final concentration of 10^5^ CFU/mL. One of the samples inoculated with sensitive *P. syringae* pv. *syringae* was inoculated with phage φ6 to obtain a final concentration of 10^5^ PFU/mL (sensitive with phage φ6) and the remaining infected sample did not have phage added (sensitive bacteria without phage). Mutants resistant to phage φ6 were added to 2 out of 4 samples to obtain a final concentration of 10^5^ CFU/mL. One of these samples was inoculated with phage φ6 (resistant bacteria with phage φ6) to obtain a final concentration of 10^5^ PFU/mL and the remaining infected sample did not have phage added (resistant bacteria without phage). Samples were incubated at 25 °C and bacterial concentration (CFU/mL) was determined in triplicate via the drop plate method in solid TSA medium at time 0 and after 6, 12, 18, and 24 h of incubation. The plates were incubated at 25 °C for 48 h. Three independent experiments were performed.

### 2.11. Assessment of the Effect of Environmental Factors upon Phage φ6 Viability

The effects of temperature, pH, and radiation (sunlight and UV-B light) upon the viability of phage φ6 (final concentration of 10^7^ PFU/mL) was tested in 30 mL of phosphate buffered saline (PBS). In the experiments, to evaluate the effect of pH and temperature, aliquots were collected after 0, 4, 6, 12, 18, 24, 30, 36, 42, 54, and 67 days of incubation. To evaluate the effect of UV-B irradiation, aliquots were collected after 0, 2, 4, 6, 8, 10, and 12 of incubation. To assess the effect of solar radiation, aliquots were collected after 0, 2, 4, and 6 h of exposure. Phage titer was determined in triplicate via the double-layer agar method and plates were incubated at 25 °C for 18 h. Three independent experiments were performed for each condition.

#### 2.11.1. pH Experiments

In order to evaluate the effect of pH upon phage viability, suspensions of phage φ6 were added to sterile PBS with pH values of 6.5, 7.0, and 7.5. During these experiments, the temperature of the samples was kept at 25 °C.

#### 2.11.2. Temperature Experiments

To evaluate the effect of temperature upon phage viability, the samples were maintained at a constant temperature (15, 25, and 37 °C) in an incubating chamber. The experiments were performed in sterile PBS at pH 7.0.

#### 2.11.3. UV-B Irradiation Experiments

In order to evaluate the effect of UV-B irradiation (290–320 nm), an ultra-violet type B lamp TL 20 W/12 RS (Philips, Holland) was used and placed at a distance of 25 cm from the samples. The experiments were performed in sterile PBS at pH 7.0 and at ambient temperature. The control sample (UV-B C) was incubated in the same conditions as the test sample (UV-B) but was not exposed to UV-B radiation.

#### 2.11.4. Solar Radiation Experiments

To evaluate the effect of solar radiation, a suspension of phage φ6 was added to sterile PBS at pH 7.0 and exposed to natural solar radiation. The control sample (SR C) was incubated in the same conditions as the test sample (S) but was not exposed to solar radiation. The experiments were performed under a solar irradiance of 2.82 kWh/m^2^ in a day with ambient temperature ranging from 14 to 24 °C.

### 2.12. Statistical Analyses

Statistical analysis of the data was performed using the software GraphPad Prism 7.04 (GraphPad Software, San Diego CA, USA). Normal distribution of the data was checked by a Kolmogorov–Smirnov test and the homogeneity of variance was assessed by the Levene’s test. The significance of bacterial and viral concentrations between treatments, and along the experiments, was tested using two-way ANOVA and the Bonferroni post-hoc test. For different treatments, the significance of differences was evaluated by comparing the result obtained in the test samples with the results obtained for the correspondent control samples, for the different times (Section 3.6). Two-way ANOVA was used to examine differences between the concentration of resistant bacteria and sensitive bacteria in the presence/absence of the phage after 6, 12, 18, and 24 h of incubation (Section 3.8). The significance of the effect of physico-chemical factors on phage φ6 viability and incubation time was assessed by one-way analysis of variance (Section 3.9). Tukey’s multiple comparison test was used for a pairwise comparison of the means. A value of *p* < 0.05 was considered to be statistically significant.

## 3. Results

### 3.1. Phage Preparation and Enrichment

**Figure 2:**
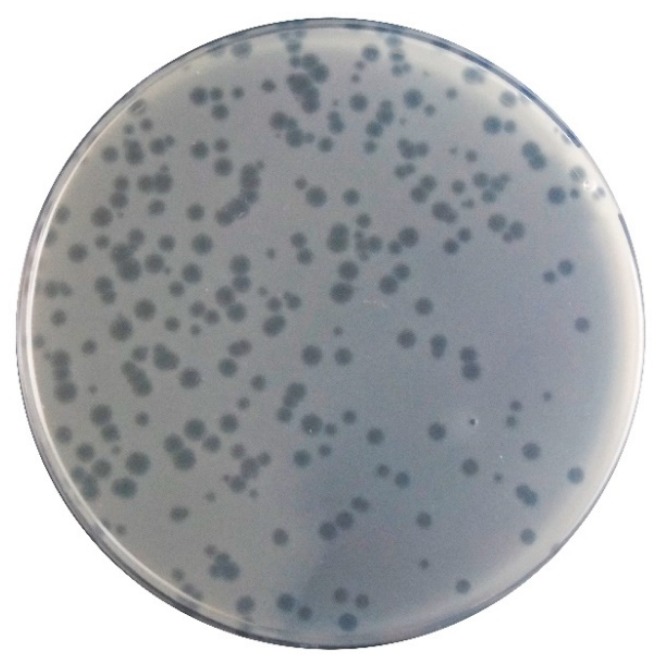
Morphology of the phage φ6 lysis plaques on its bacterial host.

Phage φ6 formed clear plaques on the *P. syringae* pv. *syringae* with a diameter of 1 to 2 mm (Figure 2). Phage plaques exhibit a secondary halo in the frontier of the lysis plaque of phage (Figure 2). High titer suspensions (10^8^–10^9^ PFU/mL) were obtained for the phage φ6.

### 3.2. Determination of the Molar Extinction Coefficient of the Isolated Phage φ6 Particles

**Figure 3:**
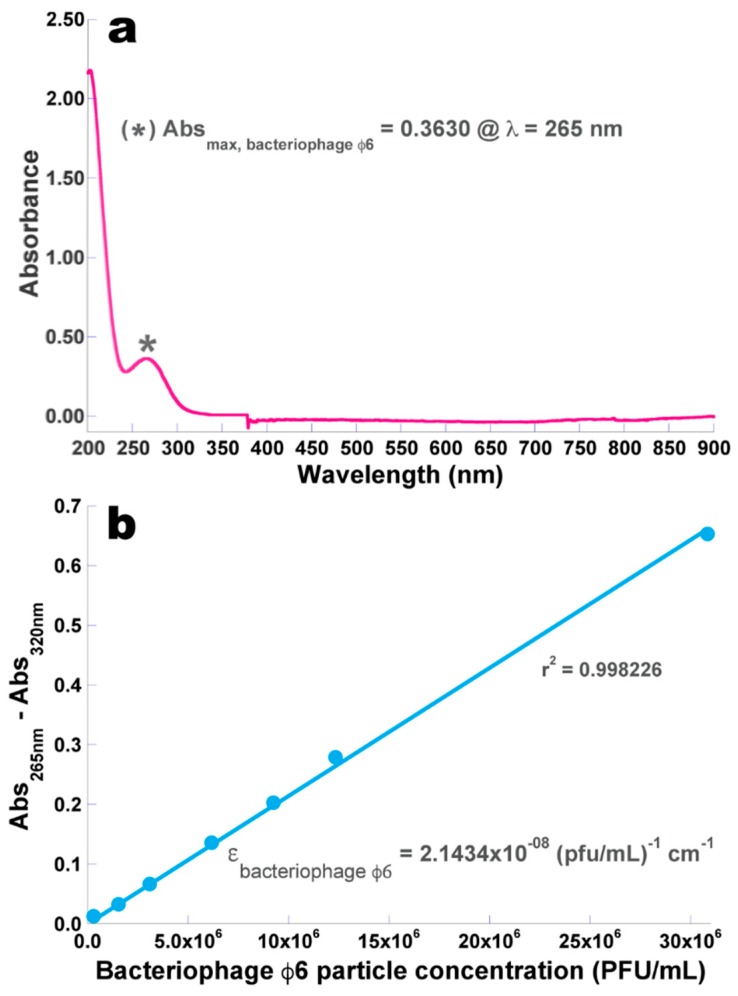
Wavelength screening of phage φ6 suspension allowing observation of the wavelength producing maximum absorption of phage φ6 particles (**a**) and the calibration curve produced for the relationship between the concentration of (whole) phage particles in suspension and the absorption of the suspension at 265 nm corrected for cell debris and other intracytoplasmic proteins at a wavelength of 320 nm (**b**).

The results obtained from the UV-Vis scanning performed to the concentrated phage φ6 suspension are displayed in Figure 3a, whereas the data utilized to prepare the calibration curve relating the phage particle concentration and its corrected absorbance (Figure 3b) are displayed in Table 2.

A pronounced minimum absorption can be observed around 245 nm (indicative of the absence of bacterial cell debris and any other intracytoplasmic proteins and the presence of high concentration of phage virions) (Figure 3a).

A linear fitting of the Beer–Lambert equation was then performed on the experimental data (Abs_265 nm_–Abs_320 nm_=ƒ (phage particle concentration, PFU/mL)), allowing determination of the molar extinction coefficient of phage φ6 as ε = 2.1434 × 10^−8^ (pfu/mL)^−1^ cm^−1^.

### 3.3. Phage Host Range and Efficiency of Plating (EOP)

The spot tests and EOP results indicated that phage φ6, in addition to *P. syringae* pv. *syringae*, formed phage lysis plaques on 2 of the 24 strains tested (Table 1). Phage φ6 infected *P. syringae* pv. *actinidiae* CRA-FRU 12.54 and *P. syringae* pv. *actinidiae* CRA-FRU 14.10, with an efficiency of 101.3% and 96.8%, respectively.

### 3.4. One-Step Growth Curve Analysis

**Figure 4:**
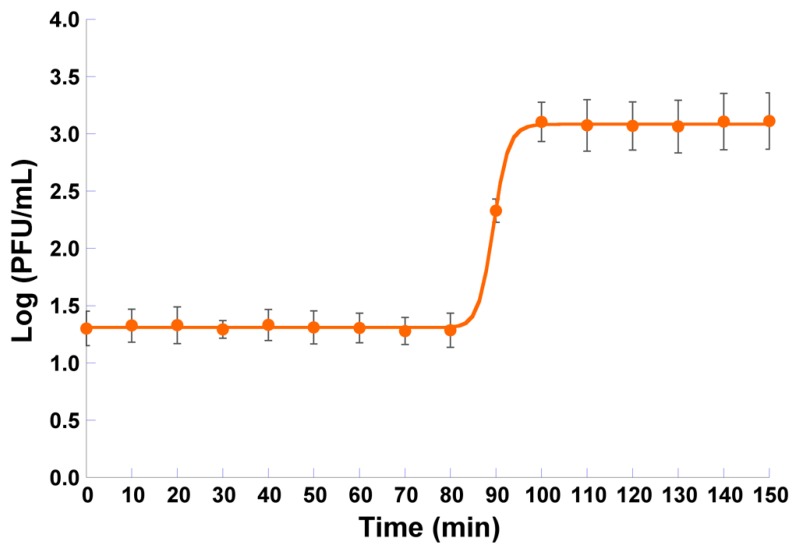
One-step growth curve of phage φ6 in the presence of *P. syringae* pv. *syringae* as the host. Values represent the mean of three experiments; Error bars represent the standard deviation.

Non-linear fitting of the one-step growth data to a four-parameter logistic model resulted in a good correlation coefficient (viz. 0.9997) and showed that the eclipse period, latent period, and intracellular accumulation period lasts 80, 100, and 20 min, respectively (Figure 4). The burst size of phage φ6 was 60 ± 1 PFU/host cell (Figure 4).

### 3.5. Adsorption Curve

**Figure 5:**
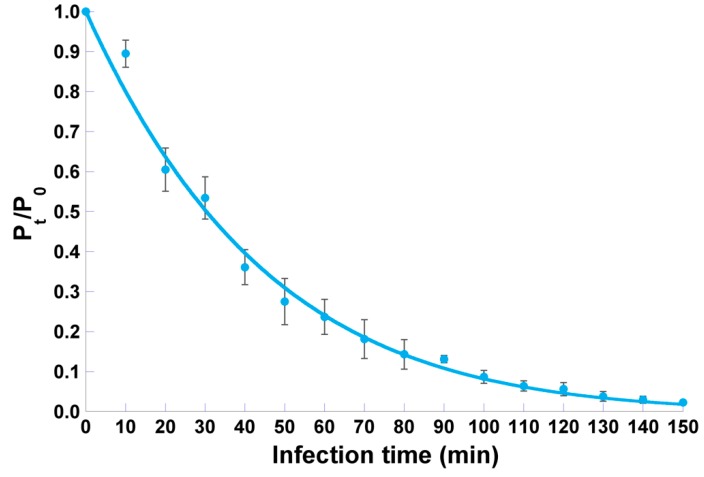
Adsorption curve of phage φ6 particles onto *P. syringae* pv. *syringae* host cells, allowing calculation of the phage particles’ adsorption rate following non-linear fitting of a logarithmic function to the experimental data. Error bars represent the standard deviation

Phage φ6 adsorption assays showed that approximately 50% of the phage particles adsorb to *P. syringae* pv. *syringae* after 30 min, 75% adsorbed after 60 min, and 95% adsorbed after 120 min (Figure 5).

Nonlinear fitting of the experimental data to the model depicted in Equation (2) resulted in a good correlation coefficient (viz. 0.9951) and allowed determination of the adsorption rate of phage φ6 onto *P. syringae* pv. *syringae* cells as δ = (9.495 ± 0.660) × 10^−12^ PFU^−1^ CFU^−1^ mL^−1^ hr^−1^ and a bacteria multiplication rate of *µ*(*t*) = (2.489 ± 2.055) × 10^−3^ hr^−1^.

### 3.6. Bacterial Kill Curves and Host Sensitivity to Phage φ6

**Figure 6:**
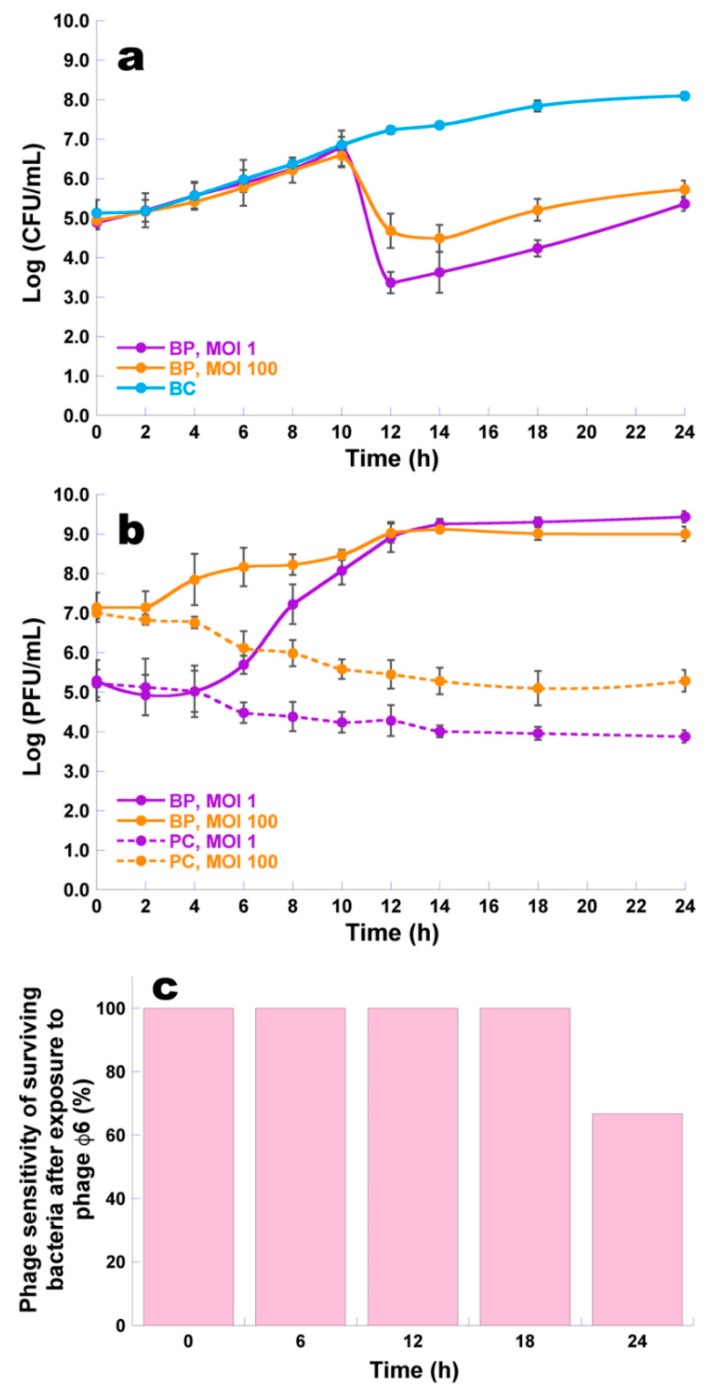
Inactivation of *P. syringae* pv. *syringae* by phage φ6 at a multiplicity of infection (MOI) of 1 and 100 during 24 h. (**a**) Bacterial concentration: BC, bacteria control; BP, bacteria plus phage; (**b**) Phage concentration: PC, phage control; BP, bacteria plus phage; (**c**) Percentage of phage sensitivity of surviving bacteria after phage exposure of *P. syringae* pv. *syringae* cells sensitive to phage φ6 at a MOI of 1, after 0, 6, 12, 18, and 24 h of incubation, following spot testing of randomly chosen bacterial colonies. Values represent the mean of three independent assays; Error bars represent the standard deviation.

At a MOI of 1 and 100, the maximum *P. syringae* pv. *syringae* inactivation with phage φ6 was 3.9 and 2.6 log CFU/mL, respectively (Figure 6a, ANOVA, *p* < 0.05), achieved after 12 and 24 h of incubation, when compared with those of the bacterial control (BC). During the first 10 h of incubation, the inactivation factor was similar for a MOI of 1 and 100 (ANOVA, *p* > 0.05). At a MOI of 1, after 12, 14, and 18 h of incubation, the decrease in *P. syringae* pv. *syringae* counts (3.9, 3.7, and 3.6 CFU/mL, respectively) was significantly higher (ANOVA, *p* < 0.05) than that obtained with the MOI of 100 (1.8, 2.1, and 2.6 CFU/mL, respectively). The rates of bacterial reduction at the end of incubation were statistically similar (ANOVA, *p* > 0.05) for the two MOI values (Figure 6a).

Bacterial density in the BC increased by 3.0 log CFU/mL (ANOVA, *p* < 0.05) during the 24 h of incubation (Figure 6a). During the 24 h timeframe of the experiments, phage concentration in the controls (PC) decreased (1.3 and 1.7 log PFU/mL, ANOVA, *p* < 0.05) for the MOI of 1 and 100, respectively (Figure 6b). When phage φ6 was incubated in the presence of its host, a significant increase in the phage particle concentration (4.1 and 1.9 log PFU/mL, ANOVA, *p* < 0.05) was observed for the MOI of 1 and 100 (Figure 6b).

At time zero and after 6, 12, 18, and 24 h of incubation, 10 colonies of the test sample were isolated, to check whether the *P. syringae* pv. *syringae* remained sensitive to phage φ6 during the bacterial kill assays. During the first 18 h of incubation, *P. syringae* pv. *syringae* was sensitive to phage φ6. However, after 24 h of incubation, it was observed that only 66.7% of the tested bacteria were sensitive to phage φ6 (Figure 6c).

### 3.7. Determination of the Frequency of Emergence of Phage-Resistant Bacterial Mutants

The frequency of *P. syringae* pv. *syringae* mutants resistant to phage φ6 was (1.20 ± 0.62) × 10^−3^ (Table 3).

### 3.8. Fitness of Phage-Resistant Mutants

**Figure 7:**
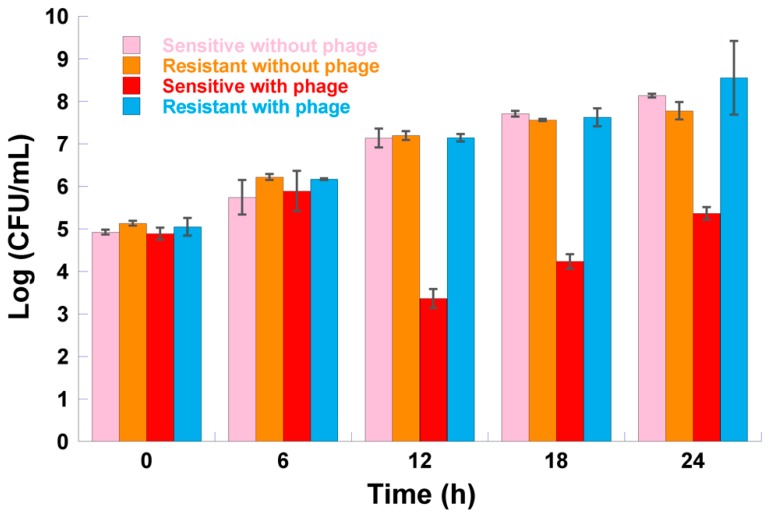
*P. syringae* pv. *syringae* concentration of resistant mutants versus their sensitive cells in the presence or absence of phage φ6 at a multiplicity of infection (MOI) of 1 after 6, 12, 18, and 24 h of incubation. Values represent the mean of three independent assays; error bars represent the standard deviation.

In the presence of phage φ6, after 6 h of incubation, no differences were found between the concentration of resistant bacteria and the concentration of sensitive bacteria (Figure 7, ANOVA, *p* < 0.05). However, after 12, 18, and 24 h of incubation, differences were observed between the concentration of resistant bacteria and the concentration of sensitive bacteria. Resistant bacteria reached a higher concentration at 12, 18, and 24 h of incubation when compared to its sensitive counterpart.

In the absence of phage φ6, no differences (Figure 7, ANOVA, *p* > 0.05) were found between the concentration of resistant bacteria and the concentration of sensitive bacteria.

During the 24 h of incubation, no differences (Figure 7, ANOVA, *p* > 0.05) were found between the concentration of resistant bacteria in the presence of phage φ6 and the concentration of resistant bacteria in the absence of phage φ6.

### 3.9. Assessment of the Effect of Environmental Factors upon Phage φ6 Viability

#### 3.9.1. Temperature Experiments

**Figure 8:**
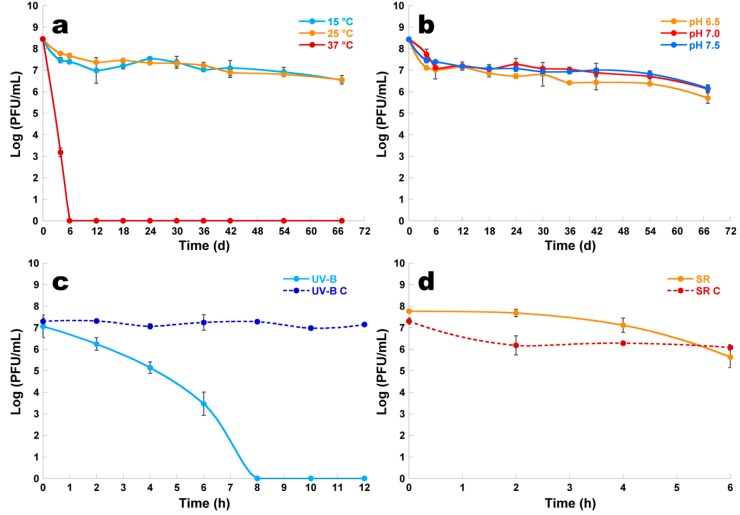
Survival of phage φ6 following exposure to different temperature values (**a**), different pH values (**b**), UV-B irradiation (**c**), and solar radiation (**d**). Values represent the mean of three independent experiments; error bars represent the standard deviation. UV-B: phage exposed to UV-B irradiation; UV-B C: phage control—phage not exposed to UV-B irradiation; SR: phage exposed to solar radiation; SR C: phage control—phage not exposed to solar radiation.

The reduction in the concentration of viable phage φ6 particles was higher at 37 °C than at 15 and 25 °C (Figure 8a, ANOVA, *p* < 0.05). A maximum decrease of 8.5 log PFU/mL was observed after 6 days when the samples were kept at a temperature of 37 °C, but after only 4 days at this temperature, the decrease was of 5.3 log PFU/mL. When the temperature was decreased to 15 and 25 °C, the rate of maximum reduction slightly decreased to 2.0 log PFU/mL after 67 days of incubation. The difference between these two temperatures was not statistically significant (Figure 8a, ANOVA, *p* > 0.05).

#### 3.9.2. pH Experiments

When different pH values (6.5, 7, and 7.5) were tested, it was observed that the phage concentration slightly decreased with the decrease of pH; however, the differences among the three values of pH were not statistically significant (Figure 8b, ANOVA, *p* > 0.05). In the three pH values studied, phage φ6 persisted as viable for at least 67 days at 25 °C (Figure 8b). The abundance of phage φ6 decreased about two orders of magnitude over the 67 days (Figure 8b, ANOVA, *p* < 0.05).

#### 3.9.3. UV-B Experiments

When phage φ6 was exposed to UV-B irradiation, it was observed that the phage concentration decreased (Figure 8c, ANOVA, *p* < 0.05) during 12 h of incubation when compared with the phage control (UV-B C). The abundance of phage exposed to UV-B irradiation decreased about 0.8 log PFU/mL after 2 h of incubation, but after 6 h the decrease was of 3.5 log PFU/mL. A maximum decrease of 7.0 log PFU/mL was observed after 8 h when compared with the phage control (UV-B C) (Figure 8c, ANOVA, *p* < 0.05) (Figure 8c). The concentration of the phage φ6 not exposed to UV-B irradiation (UV-B C) remained constant (Figure 8c, ANOVA, *p* > 0.05) during 12 h of incubation.

#### 3.9.4. Solar Radiation Experiments

When phage φ6 was exposed to solar radiation, the abundance of phage φ6 decreased 2.1 log PFU/mL (Figure 8d, ANOVA, *p* < 0.05), when compared with the phage control (SR C). The decrease in phage abundance was 0.6 log PFU/mL (Figure 8d).

## 4. Discussion

Phage φ6, one of the best-studied phages and a commercially available one, to the best of our knowledge, was only used to control infections by the plant-pathogenic *P. syringae* pv. *phaseolicola* [20]. According to our results, this phage can be used also against other *Pseudomonas syringae* pathovars, such as *P. syringae* pv. *syringae*, which are important phytopathogens for the agriculture sector because they can easily infect several horticultural plants [4,52,53,54,55,56], causing severe economic losses worldwide.

Although the major advantage of phage treatment is phage specificity, since the non-target bacterial populations should remain undisturbed, phages should be capable of lysing the majority of strains of a given bacterial species [7,14,57,58,59]. Phage φ6, besides the *P. syringae* pv. *syringae*, also infects *P. syringae* pv. *actinidiae* CRA-FRU 12.54 and *P. syringae* pv. *actinidiae* CRA-FRU 14.10 (Table 1). These results suggest that phage φ6 can be potentially used not only to control the bacterial canker and blast in *Citrus* species (such as orange and mandarin) and *Prunus* species (including sweet cherry, apricot, tart cherry, and almond) [2,3], but also to control *P. syringae* pv. *actinidiae*, which is the causal agent of the kiwifruit bacterial canker worldwide [60,61]. The effectiveness of phage φ6 was also tested in this study against other species of *Pseudomonas* and of other bacterial genera, but none of these bacteria were infected by the phage (Table 1). As the host range of phage φ6 is quite narrow, natural non-pathogenic bacteria of infected plants will not be affected by treatment with this phage. However, phage φ6 has been shown to alter its host range, mostly through mutations in its receptor binding proteins [62]. The high mutation rate associated with its RNA-based genome allow it to exploit new niches by infecting closely related *Pseudomonas* species [53,63]. Nevertheless, in the future, new phages need to be isolated and tested together with phage φ6 in order to produce a cocktail with a broader spectrum of activity to control several pathovars of *P. syringae*.

As phage φ6 is already available commercially and has been the subject of extensive genomic characterization, with its genome sequence available in the GenBank Genomes database [35], it is possible to assure at the outset that this phage is a safe biological control agent since it does not code for integrase genes nor genes coding for virulence factors and antibiotic resistance [33,34,35,36]. In addition, using the online CRISPR-CAS++ program tool (https://crisprcas.i2bc.paris-saclay.fr/CrisprCasFinder/Index) provided by Institut Pasteur (Paris, France), which enables easy detection of CRISPRs and CAS genes in user-submitted genome sequence data, no CRISPR sequences were detected in the genome of phage φ6 (obtained from https://www.ncbi.nlm.nih.gov/genome/4960?genome_assembly_id=456346).

Before the application of phages to inactivate pathogenic bacteria, it is important to characterize *in vitro* the dynamics of phage–host replication. The first step of phage infection is the attachment of the phage virion onto a susceptible bacterial host cell [64,65,66]. This adsorption process is usually described by mass-action kinetics [67], which implicitly assumes an equal influence of the host density and phage adsorption rate [48,65]. Therefore, an environment with a high bacterial host density can be considered as equivalent to a phage endowed with a high adsorption rate and vice-versa. The result obtained in this study for the phage φ6 adsorption rate (Figure 5) is in close agreement with results published by Shao and Wang (2008), Lindberg et al. (2014), and Santos et al. (2014) for *Pseudomonas* phages isolated from environmental sources [48,49,68]. The growth characteristics of phage φ6 (Figure 4) showed a relatively high burst size (60 ± 1 PFU/ host cell), indicating that phage φ6 replicates efficiently in *P. syringae* pv. *syringae* but needs a long latency period (100 min). Consequently, phage φ6 caused a high reduction in *P. syringae* pv. *syringae* growth, but its effect occurs only after 10 h of incubation.

*P. syringae* pv. *syringae* was effectively inactivated by phage φ6 (Figure 6a), reaching the maximum of inactivation of ≈4 log CFU/mL after 12 h incubation at a MOI of 1. After that, although some bacteria were not inactivated by the phage, around 3 log CFU/mL, most of the inactivated bacteria did not regrow after treatment. In fact, between 12 and 24 h of phage treatment, the bacterial concentration was significantly lower than that observed for the non-treated cultures (Figure 6a). Vidaver et al. (1973) characterized phage φ6 using the plant-pathogenic *P. syringae* pv. *phaseolicola* HB10Y, but no inactivation studies were performed by those authors. To the best of our knowledge, this phage has not yet been tested to inactivate *P. syringae* pv*. syringae* [20].

The kinetic theory of phage therapy predicts that the MOI could be critical to the efficiency of bacterial inactivation. It has been shown, both *in vitro* and *in vivo*, that the reduction of pathogenic bacteria increases in parallel with the MOI or that bacterial reduction occurs sooner at higher MOI values [69,70,71]. However, other studies [72,73,74] show that precise initial doses of phage may not be essential due to the self-perpetuating nature of phages, revealed by an increasing of phage titers along with bacteria. In the present study, the increase in MOI from 1 to 100 did not promote an increase in the efficiency of phage φ6. The number of phage particles during 24 h of incubation in the presence of the host at a MOI of 1 increased more (by 4.1 log PFU/mL) than at a MOI of 100 (by 1.9 log PFU/mL) (Figure 6b). This confirms the hypothesis that due to the self-perpetuating nature of phages, precise initial doses of phage may not be essential. In fact, this is one of the major advantages of phage treatment relative to chemical antibiotherapy. A high MOI can even be a disadvantage for the success of phage treatment since the bacteria may be inactivated before replicating the phages. This can occur when an overload of phages simultaneously infects a bacterium, leading to lysis due to the presence of high concentrations of lysins, a phenomenon known as “lysis from without” [75,76,77]. This phenomenon can explain the decrease in *Pseudomonas syringae* pv*. syringae* inactivation by the phage φ6 at MOI 100 when compared with MOI 1. In fact, at MOI 1, the increase in the phage particle number after 24 h of incubation was significantly higher (100 times higher) than that obtained when MOI 100 was used.

A major concern of bacterial inactivation by phages is the emergence of phage-resistant bacteria [7,8,43,46,51,58,77,78,79]. Phage φ6 did not prevent bacterial regrowth during treatment (Figure 6a). After 24 h of incubation, 66.7% of *P. syringae* pv. *syringae* cells were shown to be sensitive to phage φ6 (Figure 6c). However, some of the insensitive bacterial cells may be the result of not having had contact with the phage during the incubation period, not all being phage resistant. In fact, during the bacterial killing assays, it is not possible to know whether the surviving bacteria are really resistant, since the bacteria are collected from liquid medium samples (TSB), containing both phage particles and bacteria, which does not ensure that all tested bacteria were in fact in contact with the phages. The development of resistant mutants, determined only for bacteria that were in contact with the phages, was limited (1.20 × 10^−3^) (Table 3). Although these results are in close agreement with results obtained by other researchers for other *Pseudomonas* species, viz. *P. aeruginosa* strain PAO1 and phage PA5P2 (1.7 × 10^−3^) [80], the development of resistant mutants of *P. syringae* pv. *syringae* cells for phage φ6 are higher than those obtained by Rombouts et al. (2016) for *P. syringae* pv. *porri* and phage KIL3 (1.83 × 10^−6^) or phage KIL4 (3.33 × 10^−6^) [25], and those obtained by Li et al. (2018) for *P. aeruginosa* strain PA1 and phage PaP1 (3.0 × 10^−5^) [81].

Some authors have suggested that exposure to phage could cost bacteria their fitness, which can lead to their removal from the environment at a faster rate than their wild-type counterparts [82,83]. Recently, Sistrom et al. (2015) showed that *P. syringae* pv. *phaseolicola* can evolve resistance to phage φ6 by eliminating type-IV pili, but the phage mutants presented reduced virulence [53]. In our study, the experimental results of the fitness of phage showed that the concentration of sensitive bacteria and resistant mutants, when grown in the absence of phage φ6, are similar (Figure 7). Nevertheless, these experiments were carried out in nutrient-rich (culture) medium and in the absence of competition, from which, according to some authors, the cost of resistance can vary across environmental factors and the degree of competition for resources [84,85]. Meaden et al. (2015) obtained similar results for *P. syringae* pv. *tomato* under standard laboratory conditions (*in vitro*) [86]. However, when the experiments were carried out in tomato plants (*Solanum lycopersicum*), the phage-resistant bacterial mutants exhibited reduced densities relative to the sensitive bacterial population. In the future, further studies will be needed to evaluate the cost of bacterial resistance to phage φ6 in plants. Moreover, according to several authors, the resistance drawback can be overcome by the use of phage cocktails [43,46,58,79,87,88,89,90,91,92].

In order to implement this treatment in the field, it would be important to evaluate the use of phage φ6 not only as a preventive measure but also to treat active infections. The application of phage φ6 can be done by spraying the outer surface of the plants. Despite the semi-solid state nature of the plant’s tissues, as the phage particles can move in moist environments, besides infecting the bacteria that are on the surface, they can assess and control bacteria that lie within the plant’s tissues during an infection. However, there are no studies reported in the literature regarding this possibility. Although further studies are needed to test this hypothesis, the application of the phages superficially either preventively and/or to treat superficial infections by that phytopathogen seems to be an alternative option to the conventional antimicrobial treatments.

For the design and implementation of an effective phage therapy protocol to control plant diseases, the study of the stability of phages to environmental factors, such as temperature, soil pH, solar, and ultraviolet radiation, is crucial. One important factor that influences phage stability is the pH of the environment [93], influencing attachment, infectivity, intracellular replication, and amplification of phages [94,95,96]. Unfavorable pH values can interfere with the lysozyme enzyme and/or with other phage capsid proteins, thus preventing phage attachment to receptor sites on the host cell [95,96]. In this study, pH values ranging from 6.5 to 7.5 were tested (Figure 8b), which are included within the optimum neutral range of pH values for plant cultivation, and the survival of phage φ6 was not significantly affected. Generally, 10 < pH values < 5 have shown to be less efficient in studies on the lytic activity of phages, with the optimum conditions being around a neutral pH of 6 to 8 [94,97,98]. Since there is a positive correlation between soil pH and pH of fresh leaves [99,100], the range of pH values studied lie within the optimum pH range for the soil in orchards and, consequently, for the surface pH of plant leaves. This is clearly important in the context of a putative phage therapy application in the field.

Temperature is a crucial factor for phage viability in the environment [101,102], playing a fundamental role in the attachment, penetration, and amplification of phage particles in their host cells [93]. At low temperatures, only a few phages’ genetic material enters into bacterial host cells and hence fewer phage particles can be involved in the multiplication phase. On the other hand, high temperatures can promote an extended phage latency period [103]. In this study, phage φ6 was completely inactivated at 37 °C after 6 days of incubation (maximum decrease of 8.5 log PFU/mL) (Figure 8a). However, when the temperature was decreased to 15 and 25 °C, the rate of the maximum reduction in phage viability decreased to 2.0 log PFU/mL after 67 days of incubation. This means that in summer, when temperatures occasionally rise to 37 °C, bacteria may not be inactivated by the phage. However, as the most critical period for plants is autumn/winter and early spring and the infection ability of *P. syringae* pathovars at temperatures above 25 °C is reduced [104,105,106,107], temperature would not be a problem for the implementation of phage therapy.

Solar radiation or, more specifically, UV irradiation has been recognized as the most important factor for the loss of phage infectivity in the environment [73,74,108,109,110]. Solar radiation can directly affect free viruses by degrading proteins, altering structure, and decreasing infectivity [110]. Shorter wavelengths (UV-B radiation, ranging from 290 to 320 nm) impart irreversible damages to the genomic material and can result in the modification of viral proteins and formation of (lethal) photoproducts [110,111,112]. In fact, the abundance of phage φ6 particles decreased when exposed to solar radiation (decrease of 2.1 log PFU/mL after 6 h of incubation, with a solar radiation of 83.3 kWh/m^2^/day (data obtained from IPMA—Portuguese Institute of the Sea and the Atmosphere) (Figure 8c,d). Notwithstanding the fact that phage particles are sensitive to UV radiation, their sensitivity to UV wavelengths from solar radiation can be overcome by applying the phages at high titers and at the end of the day or at night, a period during which radiation is limited [74]. The phages can be applied in free form (as a spray of concentrated phage particles or cocktail of phage particles) or trapped within micro- or nanocarriers [42,113,114,115].

## 5. Conclusions

The results of this study clearly show that the commercially available phage φ6 can be a potential and effective alternative to reduce the concentration of *P. syringae* pv. *syringae*. As phage φ6 is sensitive to UV irradiation and solar radiation, they should be applied at the end of the day or during night periods, either in free form as a spray of concentrated phage particles or trapped within micro- or nanocarriers, in order to produce higher bacterial control. Nonetheless, further studies are needed, namely in the field, in order to fully understand the true potential of this phage to control infections caused by *P. syringae* pathovars.

## Figures and Tables

**Table 1:** Host range of phage φ6 determined on 25 bacterial strains. Clear lysis zone (+) and not lysis zone (−). The plating with the host strain was considered as EOP = 100%. EOP: Efficiency of Plating.

Strains	Spot Test	EOP (%)
*Pseudomonas syringae* pv. *syringae* DSM 21482	+	100 (host)
*Pseudomonas syringae* pv. *actinidiae* CRA-FRU 8.43	−	0
*Pseudomonas syringae* pv. *actinidiae* CRA-FRU 12.54	+	101.3
*Pseudomonas syringae* pv. *actinidiae* CRA-FRU 14.10	+	96.8
*Pseudomonas aeruginosa* ATCC 27853	−	0
*Pseudomonas aeruginosa*	−	0
*Pseudomonas gingeri*	−	0
*Pseudomonas putida* JQ619028	−	0
*Pseudomonas putida* JQ824856	−	0
*Pseudomonas* sp. JX047434	−	0
*Pseudomonas* sp. EF627998	−	0
*Pseudomonas* sp. AF411853	−	0
*Pseudomonas* sp. HF679142	−	0
*Pseudomonas* sp. AB772943	−	0
*Pseudomonas* sp. EU306338	−	0
*Pseudomonas* sp. AY332207	−	0
*Pseudomonas* sp. JN033360	−	0
*Pseudomonas stutzeri* EU167940	−	0
*Pseudomonas rhodesiae* JX994152	−	0
*Escherichia coli* ATCC 13706	−	0
*Escherichia coli* ATCC 25922	−	0
*Salmonella* Typhimurium ATCC 13311	−	0
*Salmonella* Typhimurium ATCC 14028	−	0
*Aeromonas hydrophila* ATCC 7966	−	0
*Vibrio parahaemolyticus* DSM 27657	−	0

**Table 2:** Data utilized to prepare a calibration curve aiming at determining the molar extinction coefficient of phage φ6 (whole) particles.

Sample Volume of Concentrated Phage Suspension (µL)	Final Volume of Dilution (µL)	Number of Phage Particles in the Sample Volume of Concentrated Phage Suspension	Phage Particle Concentration (PFU/mL)	Abs_265 nm_	Abs_320 nm_	Abs_265 nm_–Abs_320 nm_
5	3000	9.2500 × 10^5^	3.0833 × 10^5^	0.0130	0.0000	0.0130
10	3000	1.8500 × 10^6^	6.1666 × 10^5^	0.0110	0.0110	0.0000
25	3000	4.6250 × 10^6^	1.5416 × 10^6^	0.0340	0.0010	0.0330
50	3000	9.2500 × 10^6^	3.0833 × 10^6^	0.0710	0.0040	0.0670
100	3000	1.8500 × 10^7^	6.1666 × 10^6^	0.1510	0.0150	0.1360
150	3000	2.7750 × 10^7^	9.2500 × 10^6^	0.2300	0.0270	0.2030
200	3000	3.7000 × 10^7^	1.2333 × 10^7^	0.3180	0.0390	0.2790
500	3000	9.2500 × 10^7^	3.0833 × 10^7^	0.7640	0.1110	0.6530

**Table 3:** Frequency of emergence of *P. syringae* pv. *syringae* spontaneous phage-resistant mutants.

Control Sample (CFU/mL)	Sample Treated with Phage φ6 (CFU/mL)	Frequency of Phage-Resistant Bacterial Mutants
(1.47 ± 0.19) × 10^8^	(1.75 ± 0.12) × 10^5^	(1.20 ± 0.62) × 10^−^^3^
